# The Causal Effect of Iron Traits on Risk of Hypertrophic Scarring: A Two‐Sample Mendelian Randomization Study

**DOI:** 10.1111/jocd.70216

**Published:** 2025-05-02

**Authors:** Donghui Bian, Hongmin Gong, Wen Shi

**Affiliations:** ^1^ Department of Burns and Plastic Surgery The 960th Hospital of People's Liberation Army Jinan City Shandong Province China; ^2^ Department of Burns and Plastic Surgery Central Hospital Affiliated to Shandong First Medical University Jinan City Shandong Province China; ^3^ Department of Wound Repair Central Hospital Affiliated to Shandong First Medical University Jinan City Shandong Province China

**Keywords:** hypertrophic scarring, iron, iron deficiency anemia, Mendelian randomization

## Abstract

**Background:**

The involvement of iron deficiency anemia (IDA) and abnormal iron metabolism in multiple fibrotic diseases is known, but their precise relationship with hypertrophic scarring (HTS) remains uncertain.

**Aim:**

This study aimed to explore whether there are causal associations between iron traits—such as IDA, transferrin (TF), transferrin saturation (TFS), ferritin (FERR), and IRON levels—and the risk of HTS using a two‐sample Mendelian randomization (MR) approach.

**Methods:**

Relevant consortia provided genome‐wide association study (GWAS) data for iron traits, while the FinnGen study supplied GWAS data for HTS. Stringent criteria for instrumental variable (IV) selection were applied, and MR analyses were performed using the inverse‐variance weighted (IVW) method as the primary analysis, along with MR‐Egger, weighted median, and weighted mode methods. Sensitivity analyses, including the MR‐Egger intercept, Cochran's *Q* test, leave‐one‐out analysis, and MR‐PRESSO, were utilized to assess horizontal pleiotropy, heterogeneity, and outlier effects.

**Results:**

The MR analyses did not indicate significant causal links between IDA, TF, FERR, or IRON levels and the risk of HTS. While the IVW method proposed a potential protective effect of elevated TFS levels (OR = 0.69, 95% CI: 0.51–0.93, *p* = 0.0155) on HTS risk, the results varied across different MR methods. Sensitivity analyses found no significant pleiotropy or heterogeneity.

**Conclusion:**

The two‐sample MR study did not find compelling evidence for causal associations between most iron traits and HTS risk. However, the IVW method pointed to a potential protective effect of elevated TFS levels on HTS risk. Further investigation is necessary to explore the role of iron metabolism in scarring.

## 1 Introduction

Scar tissue development is an unavoidable consequence of skin repair after exposure to damaging external factors and is associated with race, gender, age, and the wound's tension, location, and injury pattern [1]. When an injury reaches the deeper reticular layer of the dermis, it may cause an abnormal fibroproliferative response, leading to hypertrophic scarring (HTS) and possibly evolving into a keloid [2]. HTS is often marked by the overproduction of collagen and an imbalance in the ratio of type I to type III collagen [3, 4]. It is estimated that around 35% of patients with postoperative scarring develop HTS within a year [5]. Beyond localized damage to the skin's appearance, scarring frequently brings along symptoms like itching and pain [6]. Mobility problems arising from severe scar contracture in functional areas can severely diminish the patient's quality of life and place considerable burdens on their life and psychological well‐being [7]. Though the precise pathophysiology remains unclear, it is hypothesized that both genetic and environmental factors contribute to the formation of HTS [8].

Essential for multiple physiological functions, iron is pivotal in processes, such as metabolism, erythropoiesis, immune function, and cognitive development [9]. There is considerable variation in systemic iron status, with serum iron showing a coefficient of variation of 30.2% in men and 36.2% in women [10]. Alterations in iron homeostasis have been suggested to contribute to the development of multiple fibrotic disorders, such as idiopathic pulmonary fibrosis, liver cirrhosis, and chronic kidney disease [11, 12]. In terms of mechanism, iron overload can drive oxidative stress, inflammation, and the activation of pro‐fibrotic signaling pathways, which in turn result in elevated extracellular matrix deposition and tissue scarring [13, 14].

Across the globe, iron deficiency anemia (IDA) prevails as the most widespread type of anemia, presenting a serious health hazard, notably for pregnant women and children [15]. Insufficient iron intake in IDA leads to diminished hemoglobin production [16]. Despite notable advancements in anemia control and management, the situation remains unsatisfactory, especially in many developing countries that lack sufficient financial and technical resources [17, 18]. Impaired wound healing and heightened risk of scarring have also been associated with IDA [19, 20]. Altered immune function, impaired collagen synthesis, and decreased angiogenesis are among the proposed mechanisms in iron‐deficient states [21, 22]. Nevertheless, the causal relationship between iron traits, such as IDA, and diverse iron biomarkers (e.g., transferrin [TF], ferritin [FERR]), and scarring remains unclear due to the possibility of confounding and reverse causation in observational studies.

Utilizing genetic variants as instrumental variables, Mendelian randomization (MR) is an analytical technique employed to explore the causal effects of exposures on outcomes, reducing the impact of confounding factors and reverse causation [23]. Through the use of genetic variants correlated with the exposure of interest (e.g., iron traits) as instrumental variables, MR analyses can yield estimates of the causal effect on the outcome (e.g., HTS) that are less influenced by confounding and reverse causation compared to conventional observational studies [24]. The objective of this study was to clarify the potential causal connections between different iron traits and the risk of HTS utilizing a two‐sample MR design and data from extensive genome‐wide association studies (GWAS).

## 2 Materials and Methods

### 2.1 Study Design

**FIGURE 1:**
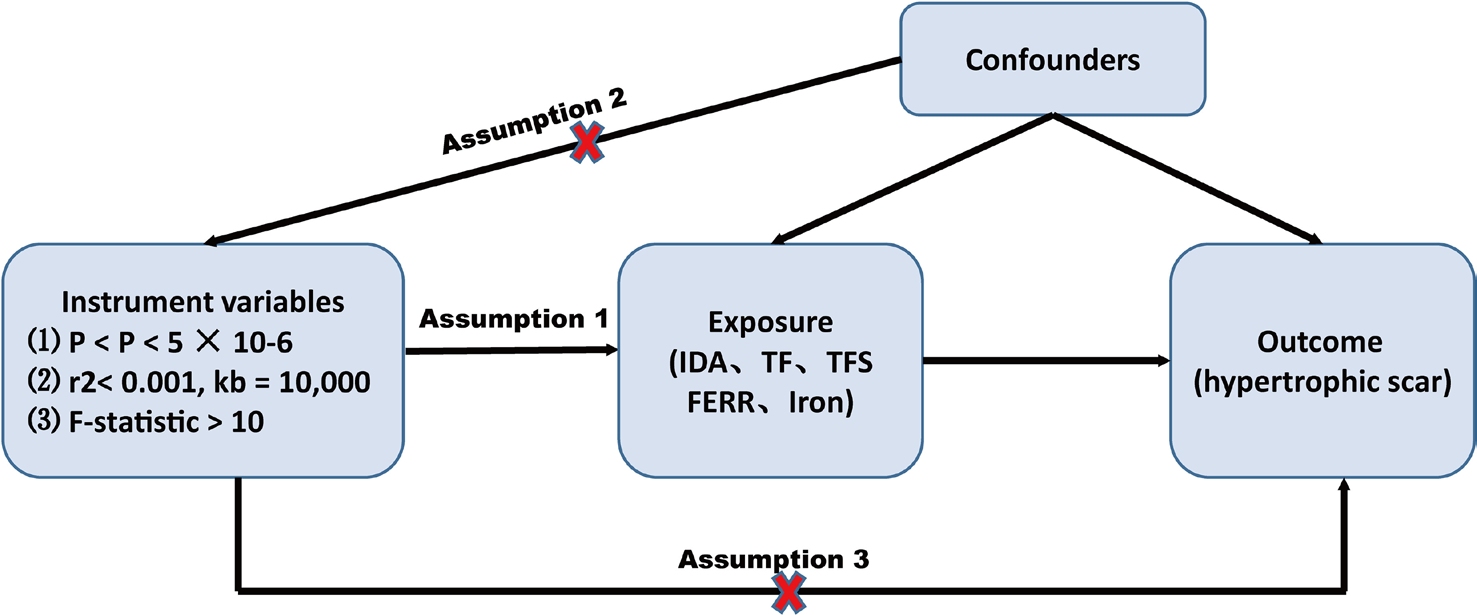
Overall study design of the MR analysis.

Using summary‐level data from distinct GWAS, we performed a two‐sample MR study with iron traits as the exposures and HTS as the outcome. This approach was configured to comply with three fundamental assumptions: (1) The IVs chosen for our study, specifically single‐nucleotide polymorphisms (SNPs), are highly correlated with the exposures; (2) The SNPs selected are not linked to any potential confounders; (3) The SNPs selected affect the outcome only by means of the exposures. The design of the MR study is overviewed in Figure 1.

### 2.2 Data Sources

The GWAS summary data on HTS were derived from the FinnGen consortium (L12_HYPETROPHICSCAR). Encompassing 16 380 443 SNPs and a sample size of 208 248 individuals from European populations, this dataset contained a vast array of information. A large summary dataset for serum iron status was retrieved from the Genetics of Iron Status consortium, and an MR analysis was performed. This dataset included 48 972 samples from European populations, collected from 11 discovery cohorts and eight replication cohorts [10]. The study encompassed iron status indicators, including serum IRON, FERR, TF, and transferrin saturation (TFS). Using data on the biochemical markers of iron status mentioned above, we conducted the MR analysis in conjunction with the IDA dataset from the FinnGen consortium (*n* = 217 202). Therefore, additional ethical approval was not required. Additional details can be found in Table 1.

**TABLE 1:** Detailed information for the GWAS data.

Trait	GWAS ID	Participants	SNPs	Population
Hypertrophic scar	finn‐b‐L12_HYPETROPHICSCAR	766/207 482	16 380 443	European
Iron deficiency anemia	finn‐b‐D3_ANEMIA_IRONDEF	6087/211115	16 380 461	European
Transferrin	ieu‐a‐1052	23 986	2 104 242	European
Transferrin saturation	ieu‐a‐1051	23 986	2 102 226	European
Ferritin	ieu‐a‐1050	23 986	2 036 124	European
Iron	ieu‐a‐1049	23 986	2 096 457	European

### 2.3 IVs Selection

Independent instrumental SNPs were utilized within the MR framework as IVs for the exposure (iron traits), enabling estimation and testing of the causal effect on the outcome (HTS). Specific criteria were applied during the selection of IVs [25]: We identified SNPs that exhibited robust associations with each trait (*p* < 5 × 10^−6^); then, independent SNPs were evaluated using pairwise linkage disequilibrium (*r* ^2^ < 0.001, kb = 10 000). The strength of individual SNPs was validated by calculating the *F*‐statistic, with a threshold set at *F* > 10 to denote the absence of significant weak instrumental bias. We assessed the strength of IVs by calculating the *F*‐statistic, employing the formula *F* = *R* ^2^ × (*N* − *K* − 1)/*K* × (1 − *R* ^2^), where *R* ^2^ signifies the proportion of variance in the exposure explained by the genetic variants, *N* represents sample size, and *K* represents the number of instruments.

### 2.4 MR Estimates

Using the R software (version 4.2.1, downloadable from https://www.r‐project.org) and the TwoSampleMR package (version 0.5.7), we carried out the two‐sample MR analyses [26]. Detection of directional pleiotropy was performed using the MR‐PRESSO package (version 1.0.0) [27]. Investigation of the causal relationship between iron traits and HTS involved the use of four methods, namely the inverse‐variance weighted (IVW), MR‐Egger, weighted mode, and weighted median. IVW was utilized for the primary MR analysis among these methods. Both the MR‐Egger method and MR‐PRESSO methods were employed to assess directional pleiotropy. The Cochran *Q*‐statistic was used to calculate heterogeneity among SNPs. To gauge the stability of the results, sensitivity analyses were employed, utilizing the leave‐one‐out sensitivity test to exclude individual SNPs one by one [28]. We determined causal estimates between IVs and outcomes by computing an odds ratio (OR) along with its 95% confidence interval (CI).

## 3 Results

### 3.1 Genetic Instruments Selection

During the MR analysis, this study employed IDA, TF, TFS, FERR, and IRON as exposures, resulting in the selection of 14, 23, 13, 14, and 11 corresponding IVs. Subsequently, after IV calculation, the mean *F*‐statistics were determined to be 24.92, 104.87, 98.23, 29.03, and 68.81, with minimum values of 18.05, 19.2, 18.45, 18.02, and 17.54, and maximum values of 76.03, 1158.71, 684.08, 109.13, and 283.67. For each exposure, 0, 0, 0, 1, and 0 SNPs did not match information in the summary data, respectively. Details regarding the number of SNPs are provided in Tables S1–, S5.

### 3.2 MR Analysis

**FIGURE 2:**
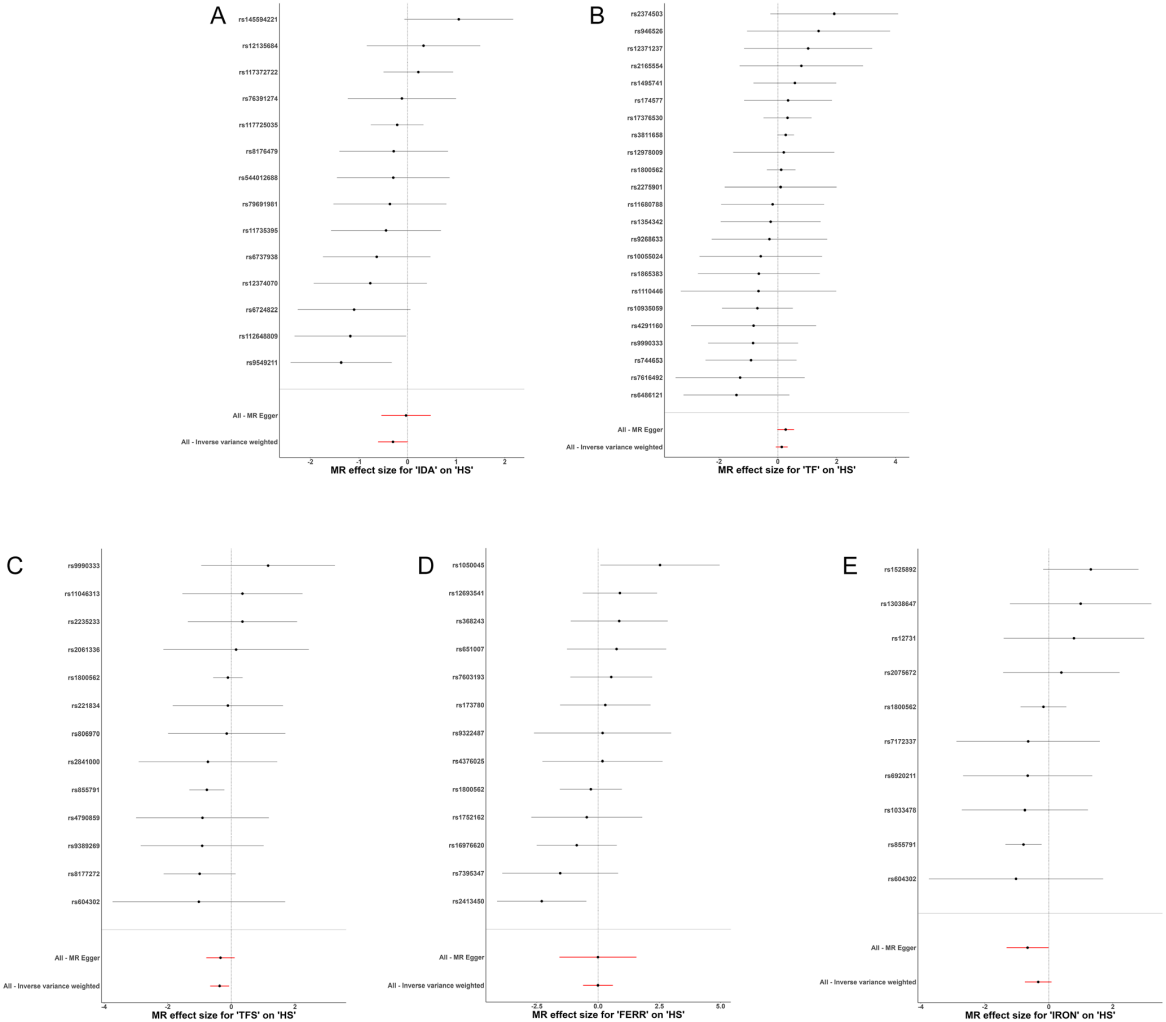
Forest plots that estimate the causal associations between IDA(A), TF (B), TFS (C), FERR (D), IRON (E) and hypertrophic scarring by using two‐sample MR analysis.

**FIGURE 3:**
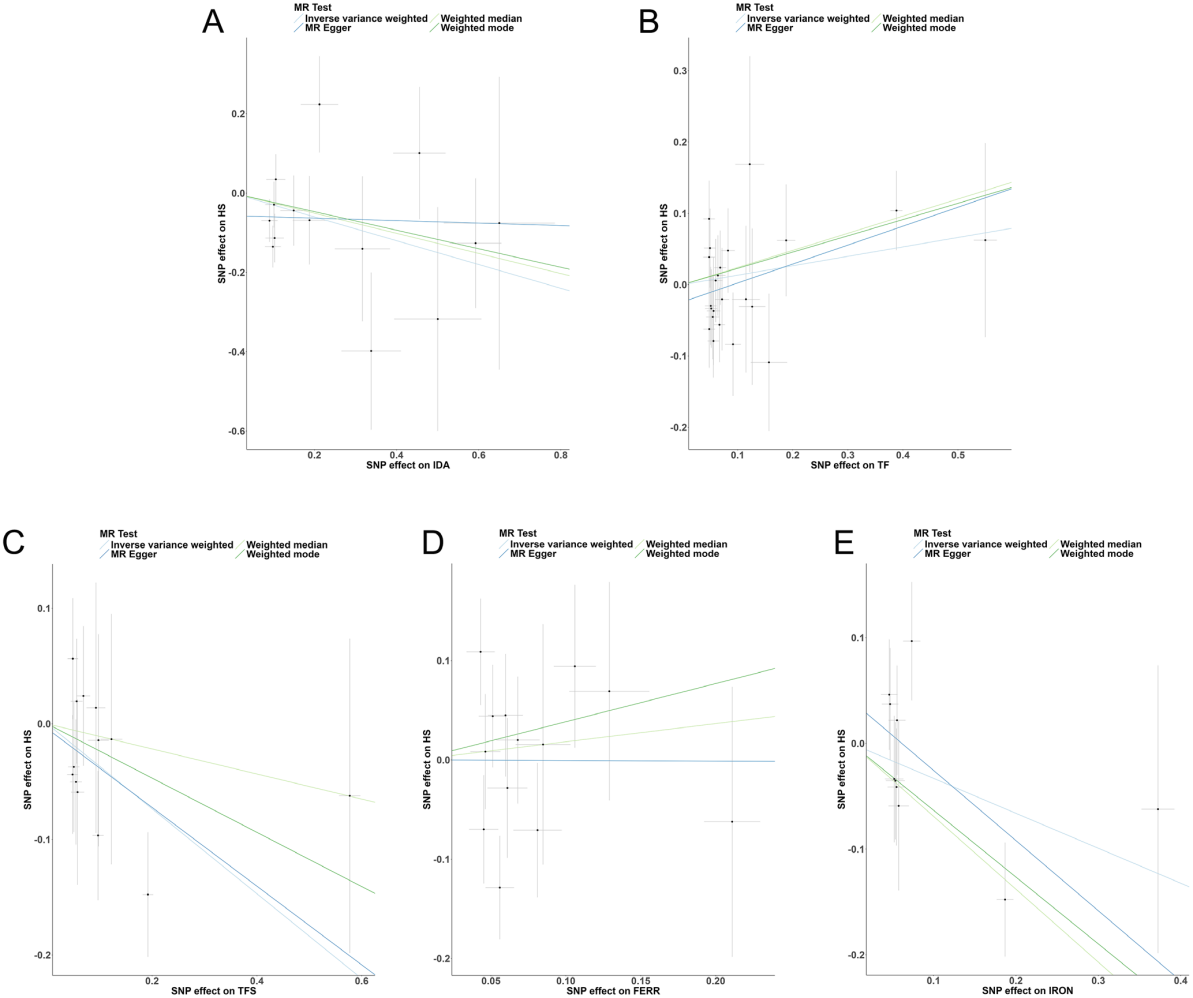
Scatter plots of the causal relationships between IDA (A), TF (B), TFS (C), FERR (D), IRON (E) and hypertrophic scarring. The slope of each line corresponds to the causal estimates for each method. Individual SNP effect on the outcome (point and vertical line) against its effect on the exposure (point and horizontal line) was delineated in the background.

As shown in Table 2 and Figure 2, the MR analysis did not uncover significant causal links between IDA, TF, FERR, and IRON levels and the risk of HTS. However, the IVW method hinted at a potential protective effect of increased TFS (OR = 0.69, 95% CI: 0.51–0.93, *p* = 0.0155) against HTS, although inconsistencies were observed across different MR techniques. Visualization of the estimated effect sizes for SNPs of iron traits on HTS was done using a scatter plot (Figure 3).

**TABLE 2:** Association between iron traits and hypertrophic scarring.

Exposure	Outcome	N.SNPs	Methods	OR (95% CI)	*p*
IDA	HTS	14	IVW	0.74 (0.54–1.01)	0.0557
			MR‐Egger	0.97 (0.58–1.61)	0.9056
			Weighted median	0.78 (0.52–1.16)	0.2209
			Weighted mode	0.79 (0.48–1.3)	0.3722
TF		23	IVW	1.14 (0.93–1.4)	0.1974
			MR‐Egger	1.3 (0.98–1.73)	0.0802
			Weighted median	1.27 (0.99–1.64)	0.0613
			Weighted mode	1.26 (0.99–1.59)	0.0699
TFS		13	IVW	0.69 (0.51–0.93)	0.0155
			MR‐Egger	0.71 (0.46–1.11)	0.1621
			Weighted median	0.9 (0.58–1.39)	0.6289
			Weighted mode	0.79 (0.5–1.24)	0.3285
FERR		13	IVW	0.99 (0.54–1.82)	0.9854
			MR‐Egger	0.99 (0.21–4.78)	0.994
			Weighted median	1.2 (0.56–2.58)	0.6391
			Weighted mode	1.47 (0.48–4.53)	0.516
IRON		11	IVW	0.72 (0.47–1.09)	0.1191
			MR‐Egger	0.51 (0.27–0.99)	0.0824
			Weighted median	0.5 (0.29–0.85)	0.011
			Weighted mode	0.53 (0.32–0.87)	0.0329

### 3.3 Sensitivity Analysis

**FIGURE 4:**
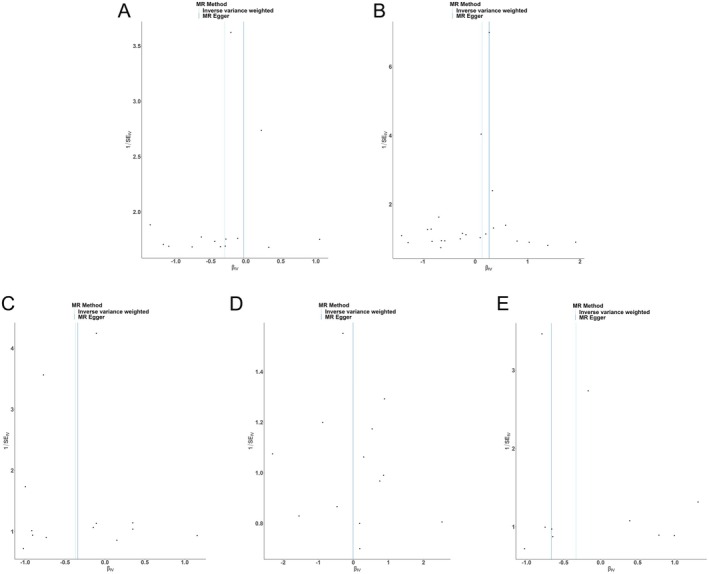
Funnel plots were applied to detect whether the observed associations between IDA (A), TF (B), TFS (C), FERR (D), IRON (E) and hypertrophic scarring, were along with obvious heterogeneity.

**FIGURE 5:**
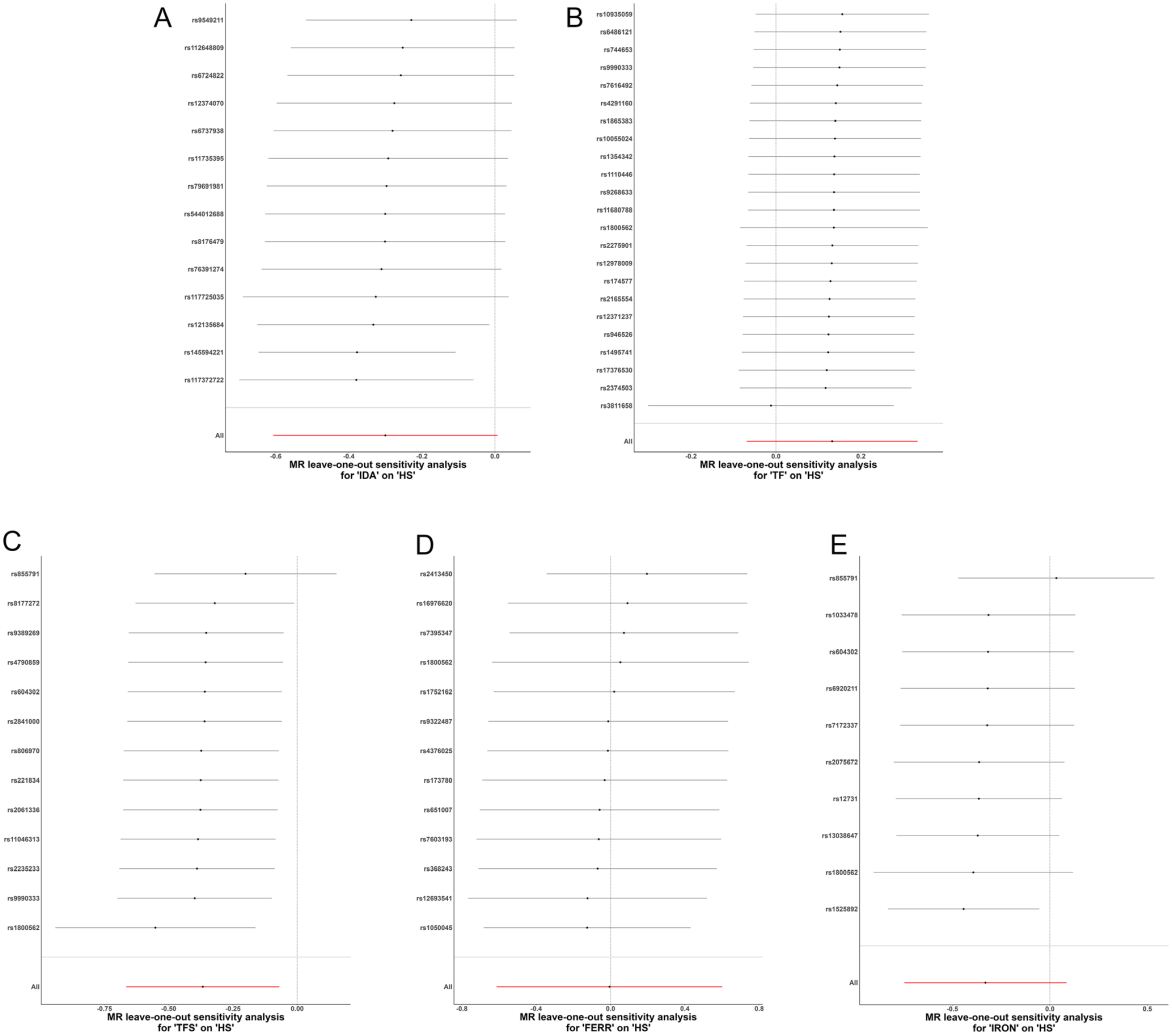
Leave‐one‐out sensitivity analysis of iron traits on hypertrophic scarring. (A) IDA; (B) TF; (C) TFS; (D) FERR; (E) IRON.

As indicated in Table 3, the MR‐Egger intercept test did not reveal significant directional pleiotropy for any of the iron traits. Additionally, Cochran's *Q* statistic did not suggest substantial heterogeneity in the IVW estimates (Figure 4). The leave‐one‐out analysis, which iteratively removes one IV at a time, did not find any single SNP driving the observed MR estimates (Figure 5). Moreover, the MR‐PRESSO global test did not detect any significant outlier SNPs, and the distortion test results were nonsignificant, indicating that the MR findings were not unduly influenced by potential outliers or horizontal pleiotropy (Table 4).

**TABLE 3:** Results of heterogeneity test and pleiotropy test of instrumental variables.

Exposure	Outcome	Heterogeneity		Pleiotropy	
		*Q* statistic (IVW)	*p*	MR‐Egger intercept	*p*
IDA	HTS	18.128	0.153	−0.057	0.222
TF		18.392	0.683	−0.024	0.204
TFS		8.933	0.709	−0.004	0.876
FERR		16.482	0.17	0	1
IRON		11.015	0.275	0.041	0.242

**TABLE 4:** MR‐PRESSO test results.

Exposure	Outcome	Raw		Outlier corrected		Global *p*	Number of outliers	Distortion *p*
		OR (CI%)	*p*	OR (CI%)	*p*			
IDA	HTS	0.74 (0.54–1.01)	0.08	NA	NA	0.184	NA	NA
TF		1.14 (0.95–1.37)	0.17	NA	NA	0.669	NA	NA
TFS		0.69 (0.54–0.9)	0.02	NA	NA	0.626	NA	NA
FERR		0.99 (0.54–1.82)	0.99	NA	NA	0.198	NA	NA
IRON		0.75 (0.5–1.12)	0.19	NA	NA	0.231	NA	NA

## 4 Discussion

In this two‐sample MR study, we explored potential causal relationships between various iron traits, including IDA, TF, TFS, FERR, and IRON levels, and the risk of HTS. Our results did not strongly support causal associations between most of the examined iron traits and HTS formation. Nevertheless, there were suggestive findings of a potential protective effect of increased TFS, indicating the necessity for additional investigation.

Contrary to observations hinting at a connection between iron deficiency and compromised wound healing or elevated scarring risk, our study did not find a significant causal effect between IDA and HTS [19, 29]. The results of earlier studies might have been impacted by confounding factors such as malnutrition, chronic inflammation, or underlying comorbidities, which could affect both iron status and wound healing processes. Yet, the MR approach applied in our study is less vulnerable to such confounding, as genetic variants are randomly distributed during gamete formation and are not usually associated with conventional confounders [30].

Our study findings did not endorse a causal relationship between circulating TF or FERR levels and HTS development. TF serves as the primary iron transport protein in the body, whereas FERR functions as the major iron storage protein [31]. Despite dysregulation of these proteins being linked to various fibrotic disorders, such as liver fibrosis and idiopathic pulmonary fibrosis [11, 32], our results suggest that their influence may not extend to the pathogenesis of HTS.

The IVW method revealed an intriguing insight, suggesting a potential protective effect of increased TFS on the risk of HTS, despite inconsistencies across different MR methods. These findings are consistent with the proposed role of iron deficiency in compromising wound healing and collagen synthesis [33, 34]. Collagen production, angiogenesis, and immune function, all critical processes in wound repair, depend on the essential role of iron in cellular processes [35, 36]. This can lead to delayed or dysregulated wound healing, potentially contributing to excessive scarring, as iron deficiency may impair these processes [37, 38]. That said, the inconsistency among all MR methods and the relatively wide confidence intervals indicate the importance of exercising caution in interpretation and highlight the need for further investigation in larger studies.

It is crucial to recognize the limitations of our study. First, despite the two‐sample MR design utilizing extensive GWAS datasets to mitigate concerns about low statistical power, it remains susceptible to potential sample overlap between the exposure and outcome datasets. Second, our analysis was narrowly focused on HTS, and the outcomes may not be broadly applicable to other types of abnormal scarring, such as keloid formation or atrophic scarring. Third, our utilization of summary‐level GWAS data may have restricted our capability to undertake more advanced analyses or stratify by relevant subgroups. Finally, our study solely examined the causal effects of iron traits on HTS risk, without exploring the potential mechanisms underlying these relationships. Additional experimental and mechanistic studies are needed to clarify the specific roles of iron homeostasis in the pathogenesis of HTS.

In conclusion, this two‐sample MR study found limited evidence for causal relationships between genetically predicted iron traits and HTS risk. Despite the IVW method suggesting a potential protective effect of increased TFS, findings were inconclusive across different MR methods. Additional research, including larger GWAS studies and complementary experimental approaches, is necessary to better understand the involvement of iron metabolism in scarring processes and explore potential therapeutic interventions.

## Author Contributions

Donghui Bian and Hongmin Gong carried out the studies, participated in collecting data, and drafted the manuscript. Donghui Bian and Wen Shi performed the statistical analysis and participated in its design. Donghui Bian, Hongmin Gong, and Wen Shi participated in the acquisition, analysis, or interpretation of data and drafted the manuscript. All authors read and approved the final manuscript.

## Ethics Statement

The authors have nothing to report.

## Consent

The authors have nothing to report.

## Conflicts of Interest

The authors declare no conflicts of interest.

## Supporting information


**Table S1.** The results of SNP selection (IDA to HTS).

**Table S2.** The results of SNP selection (TF to HTS).

**Table S3.** The results of SNP selection (TFS to HTS).

**Table S4.** The results of SNP selection (FERR to HTS).

**Table S5.** The results of SNP selection (IRON to HTS).

## Data Availability

All data generated or analyzed during this study are included in this published article.
